# Overlooked Bias with Thermometer Evaluations Using Quickly Retaken Temperatures in EHR: Axillary, Oral, Temporal Artery, and Tympanic Thermometry

**DOI:** 10.1007/s11606-021-06930-2

**Published:** 2021-06-02

**Authors:** Charles Harding, Marybeth Pompei, Dmitriy Burmistrov, Francesco Pompei

**Affiliations:** 1 Seattle, WA USA; 2 Exergen, Corp., Watertown, MA USA; 3 Woburn, WA USA

To the Editors:

Recently, Haimovich et al.^1^ sought to inform COVID-19 screening practices by evaluating temporal artery (forehead) thermometers (TATs). They retrospectively searched electronic health records (EHR) for temperatures measured twice within 15 min, including once with a TAT. The TAT often disagreed with a reference measurement, so Haimovich et al. concluded TATs perform poorly. To examine the validity of Haimovich et al.’s analysis, we extended it to the other major, non-invasive thermometer types: oral, tympanic, and axillary.

The eICU Collaborative Research Database v2.0 provides deidentified EHR from 2014 to 2015, including 139,367 intensive care unit patients at 204 US hospitals.^2^ All study data^2^ and code^3^ are available online. In this retrospective, observational analysis, we studied 154 hospitals with ≥50 chart records listing studied thermometer sites. Excluding 4852 patients aged <18 years and 1431 without temperature records, 108,970 adult patients and 5,304,829 temperatures were available. We excluded 30.4% of temperatures because a studied thermometer site was not listed, 0.3% as implausible (<50°F or >120°F), and 104 as potential double entries. From the remaining 3,670,376 temperatures and 99,858 patients, we identified measurements retaken within 15 min: 160,130 matched temperatures (4.3%) from 24,765 patients.

Axillary, central, oral, temporal, and tympanic sites were studied. Each has strengths and weaknesses in terms of accuracy, safety, and convenience.^4^ Central temperature was defined as pulmonary artery, urinary bladder, esophageal, rectal, or core (subtype unspecified). Pulmonary artery is the only true gold standard,^5^ but is highly invasive and rarely taken (0.6% of temperatures), so all central temperatures were used as a reference standard instead. TATs were hospital-grade, skin-contacting thermometers, which have a markedly different performance from the non-contact thermometer guns common since COVID-19.^6^ In EHR, thermometer site was recorded as text, which we categorized into thermometer site groups for entries appearing ≥10 times, amounting to 99.98% of entries.

We evaluated agreement between paired temperatures using Bland-Altman analyses. For measurements retaken repeatedly within 15 min, we included the first 2 temperatures only (*n*=160,130). Alternatively, one could average temperatures taken at the same site within 15 minutes,^1^ but that would make values less noisy for sites retaken more frequently, biasing Bland-Altman results. Analyses were cluster bootstrapped by patient to address within-patient nonindependence (replicates=20,000). Re-running analyses with only 1 measurement pair per patient also produced similar results.

Patients whose temperatures were retaken within 15 min (*n*=24,765) had mostly similar characteristics to the overall patient population (*n*=99,858): 44.2% and 45.4% women, mean ages 65 and 64 years, 21.6% and 20.2% with diabetes, and 14.1% and 11.7% with admission diagnosis of sepsis. Paired temperatures (*n=*160,130) were 12.6% axillary, 45.4% central, 29.2% oral, 10.2% temporal, and 2.6% tympanic.

**Figure 1:**
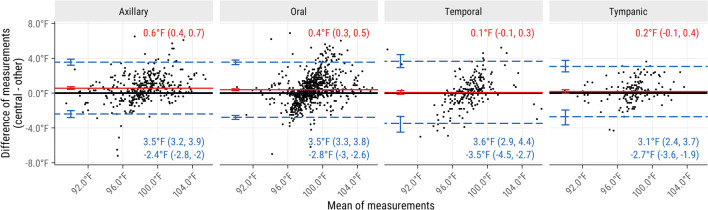
Bland-Altman comparison of central temperatures with temperatures taken at other sites within 15 min. Data are from adult critical care patients. All thermometer sites show similar, poor levels of agreement with central temperatures, though mean differences from central temperatures appear larger for axillary and oral thermometers than for temporal and tympanic thermometers. Confidence intervals are 95%. Mean differences (mean biases) are shown in red and limits of agreement (mean difference ± 2 standard deviations) are shown in blue. Central temperatures include pulmonary artery, esophageal, urinary bladder, rectal, and core.

**Figure 2:**
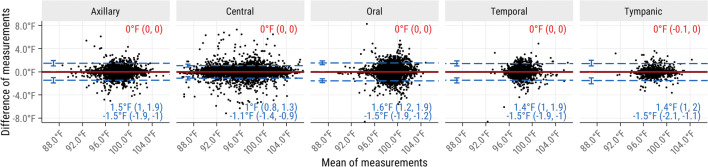
Bland-Altman comparison of temperatures taken twice at the same site with 15 min. The results show poor repeatability for all temperature sites, suggesting that a major reason for remeasurement may have been suspected clinician or patient error during a measurement. Additionally, measurements taken at central sites often reached low values that are physiologically rare, but common for measurement technique errors. As previously, data are from adult critical care patients, mean differences are shown in red, limits of agreement are shown in blue, and confidence intervals are 95%.

Every thermometer site had similar, very low agreement with reference temperatures (Fig. 1). No site satisfied the ±0.9°F (±0.5°C) limits of agreement often used to define clinical acceptability.^5^ When analyzing temperatures retaken at the same site, every site showed unprecedentedly low repeatability (Fig. 2). Retaken central temperatures were often anomalously cold (9.9% <95°F and 4.2% <92°F).

In summary, quickly retaken temperatures have very low repeatability and agreement in EHR, to a degree unprecedented by previous, non-EHR research on thermometer performance.^4,5^ Haimovich et al.^1^ observed this for TATs and we observed that it was equally the case for oral, tympanic, and axillary thermometers too. Anomalously cold central temperatures were also common in both studies.^1^

A natural explanation is that clinicians often retook temperatures for the same reason people often retake other measurements—because they suspect a measurement was done incorrectly. Common errors during temperature measurement include patient movement, accidental button presses, and insufficiently deep rectal or esophageal probes. This last error produces artificially cold temperatures, but correcting it discomforts patients, so clinicians sometimes try non-invasive thermometers instead.

This explanation means the retaken EHR temperatures likely include disproportionate numbers of errors. They are therefore invalid for evaluating usual thermometer accuracy, precision, and fever detection capabilities, and also invalid for suggesting revised COVID-19 fever thresholds (which Haimovich et al. did). Our analysis provides an illustrative example of unexpected biases that can affect EHR-based research. To detect similar problems in other EHR-based studies, researchers should place themselves in the clinicians’ shoes and carefully consider why actions listed in EHR were taken.
